# Recommendations for Writing Clinical Research Manuscripts: From Monofocal to Multifocal Intraocular Lenses

**DOI:** 10.3390/ijerph192417036

**Published:** 2022-12-19

**Authors:** Joaquín Fernández, Carlos Rocha-De-Lossada, Manuel Rodríguez-Vallejo

**Affiliations:** 1 Ophthalmology Department, Qvision, VITHAS Almería, 04120 Almería, Spain; 2 Ophthalmology Department, VITHAS Málaga, 29016 Málaga, Spain; 3 Hospital Regional Universitario de Málaga, Plaza del Hospital Civil, S/N. 29009 Málaga, Spain; 4 Departamento de Cirugía, Área de Oftalmología, Universidad de Sevilla, Doctor Fedriani, S/N, 41009 Sevilla, Spain

Writing a manuscript is not an easy task, and publishing in peer-reviewed journals might prove difficult if the methodology is not appropriately described and results are not clearly presented. Fortunately, researchers have tools that can assist in creating a well-written manuscript. For example, reporting guidelines are used by researchers to write a manuscript. These specify the minimum amount of information necessary for a clear account of research methods and findings to prevent the common pitfalls that investigators may experience during manuscript writing. The EQUATOR network manages an important database (https://www.equator-network.org/ (accessed on 15 December 2022) containing universal guidelines depending on study design and regardless of study topic [1].

However, for specific topics, such as for intraocular lens (IOL) clinical studies, merely following reporting guidelines may not be enough to produce a well-written manuscript. For example, although researchers can follow the CONSORT checklist for reporting the results of a randomized clinical trial, this checklist is focused on general characteristics; therefore, important points for a specific topic may go unnoticed by the researcher during the writing process. To complement these general guidelines, exploring specialized standards for a particular topic can improve manuscript writing. Regarding IOL clinical studies, standards for reporting clinical results with monofocal IOLs were coined in 2017 [2], and recently these have been updated for presbyopia-correcting IOLs, namely enhanced monofocal, extended depth-of-focus (EDOF), and multifocal IOLs [3]. Furthermore, standards have been defined for assessing prediction errors in spherical and toric IOL power calculation methods [4,5]. All these standards should be reviewed together with the universal reporting guidelines to create a well-written manuscript. In this editorial, we also provide a checklist (Table 1) that extends and complements the previously described guidelines and standards.

## Figures and Tables

**Table 1:** Checklist of items recommended to be included in reports of IOLs. (v1.0; December 2022).

	Item No	Recommendation	Page No
Title	1	(a) Include the keyword “intraocular lens” and the classification of the lens (e.g., extended depth-of-focus intraocular lens);	
		(b) If the study is focused on a specific population, include the corresponding keyword (e.g., cataract, refractive lens exchange, post laser refractive surgery, etc.).	
Abstract	2	(a) Include the intraocular lens/es name (model), e.g., Tecnis Eyhance (ICB00); (b) List the measured endpoints or outcomes (e.g., visual acuity, defocus curve, etc.) and the follow-up.	
Methods			
Setting	3	(a) Describe the center where the study was conducted and the period of time (e.g., at Qvision, Almería, Spain, between July 2021 and May 2022); (b) Describe whether the recruitment was consecutive (all patients were offered to participate during this period) and include recruitment loss (patients who withdrew from the study before completion).	
Participants	4	(a) Clearly state the number of eyes and patients implanted with each IOL and if this was bilateral with the same lens in both eyes, simultaneously conducted or spaced in time (including time space), and if follow-up time was defined from the first or second eye surgery; (b) Define the characteristics of the sample: cataract, refractive lens exchange, and others; (c) Describe the inclusion and exclusion criteria, including the cut-off for corneal astigmatism, exclusion of patients with surgical complications, comorbidities, etc.	
Intraocular lens	5	Explain the IOL design, optics, material, and platform, or cite any references that contain this information.	
Variables	6	(a) Define the instruments or devices used for all variables included in the results: device for pupil diameter measurement, biometer, visual acuity chart for each distance, etc.; (b) Describe the test luminance background (e.g., 85 cd/m^2^) and environmental light conditions (e.g., 100 lx); (c) For the study endpoints, measurement follow-ups should be clearly stated, in addition to the testing distances (e.g., 4 m, 66 cm, and 40 cm for far, intermediate, and near, respectively), if endpoints were measured with best or without best distance correction, and whether measurements were monocular or binocular. Additionally, do not forget the same for secondary outcomes, e.g., defocus curves, contrast sensitivity, etc.; (d) For tests presented at a different distance from that aimed to be evaluated, describe the lens power inserted for vergence correction (e.g., contrast sensitivity test presented at 2 m and aimed to report far-distance contrast sensitivity, +0.5 D lens inserted); (e) Secondary outcomes should also be precisely described. For instance, for defocus curves, indicate whether any technique to prevent the memorization of letters was applied. For the contrast sensitivity function (in log10 units), describe if the patient could not see any patch, if the bottom patch value was selected for the statistical analysis.	
Surgery	7	(a) Number of surgeons that participated in the implantation; (b) Include the incision size, location (e.g., steepest meridian, temporal, etc.), and the nucleus fragmentation type (phaco or femto), the additional incisions (e.g., arcuates, paired, etc.), nomogram, and capsulorrhexis diameter; (c) Define the formula used for selecting the IOL implant power, the constant, and if the target was programmed for emmetropia.	
Statistical methods	8	(a) Define if one (right, left, or random) or both eyes were included in the descriptive or/and inferential monocular analysis.	
Results			
Participants	9	Report the numbers of individuals/eyes finally included in the analysis. For those recruited and not included in the analysis, explain the reasons.	
Descriptive data and confounding	10	Include a table with the demographic characteristics of the sample and the biometric characteristics of the eyes (age, sex; Km, ACD, AXL, corneal astigmatism, IOL power, pupil diameter, target, etc.). If more than one group is compared, conduct inferential statistics for differences (confounding factors).	
Safety	11	(a) List the adverse events or complications, excluding those for which the endpoints can be affected (outliers), and reflect the particular endpoint value achieved for these cases; (b) Describe the % of eyes that lost postoperative CDVA in comparison with preoperative CDVA (≥0.2 logMAR) and the % of eyes that achieved a CDVA ≥ 0.3 logMAR; (c) Report the % of eyes that required Nd-YAG during follow-up.	
Efficacy	12	(a) Include a table with the descriptive statistics for postoperative-measured visual acuities; (b) Include the standard plots of efficacy with the cumulated % of eyes achieving a particular value of visual acuity [3].	
Predictability	13	(a) Report the distance at which the patient was subjectively refracted and explain if this subjective refraction was corrected to infinity or 6 m; (b) Present the postoperative subjective refraction relative to the intended target (prediction error) and the standard plot [3]. (c) Present the descriptive statistics for the postoperative subjective astigmatism and the standard plot [3].	
Secondary outcomes	14	Other secondary endpoints can be optionally included. Especially important are defocus curves; therefore, include the standard plots for defocus curves and the contrast sensitivity function with error bars [3].	
Patient reported outcomes	15	Important questions to be answered in studies with IOLs are spectacle independence, satisfaction without spectacles, positive dysphotopsia rates, and the decision to be implanted with the same IOL. For any of the previously evaluated domains using a Likert scale of four or five possible answers, include the % of subjects cumulated for the last two levels (i.e., % satisfied or very satisfied).	

These recommendations are an extension and do not replace the study-type guidelines (https://www.equator-network.org/ (accessed on 15 December 2022) or the standards for collecting and reporting results [3].
